# Resting Muscle Shear Modulus Measured With Ultrasound Shear-Wave Elastography as an Alternative Tool to Assess Muscle Fatigue in Humans

**DOI:** 10.3389/fphys.2019.00626

**Published:** 2019-05-21

**Authors:** Julien Siracusa, Keyne Charlot, Alexandra Malgoyre, Sébastien Conort, Pierre-Emmanuel Tardo-Dino, Cyprien Bourrilhon, Sebastian Garcia-Vicencio

**Affiliations:** ^1^Unité de Physiologie de l’Exercice et des Activités en Conditions Extrêmes, Département Environnements Opérationnels, Institut de Recherche Biomédicale des Armées, Brétigny-sur-Orge, France; ^2^Antenne Médicale d’Orléans-Bricy, Bricy, France

**Keywords:** isometric contractions, exercise, SWE, ultrasonography, peripheral fatigue, muscle function, muscle compliance

## Abstract

The aim of this study was to investigate the time course of the resting *vastus lateralis* (VL) muscle shear elastic modulus (μ) measured with ultrasound shear-wave elastography during repetition of isometric maximal voluntary contractions (MVCs) of the knee extensors (KE). Fifteen well-trained young males repeated 60 5-s isometric MVCs. Evoked electrical stimulations and the VLμ were measured every ten MVCs at rest. The resting VLμ significantly decreased (−34.7 ± 6.7%; *P* < 0.001) by the end of the fatigue protocol. There was also a 38.4 ± 12.6 % decrease in MVC after exercise (*P* < 0.001). The potentiated doublet and single twitch torque amplitudes and properties were significantly modified by the end of exercise (*P* < 0.001). This study shows the time course of the resting VLμ during the repetition of maximal voluntary fatiguing exercise of the KE muscles. The decrease of the resting VLμ could directly affect the force transmission capabilities accounting for peripheral fatigue.

## Introduction

Neuromuscular fatigue is classically defined as “an exercise-induced reduction in the ability of skeletal muscle to produce power or force, irrespective of task completion” (Gandevia, 2001). Potential factors involved in muscle fatigue were classified into two categories, i.e., (i) central factors involving the central nervous system and neural pathways, and (ii) peripheral factors occurring within the muscle, beyond the neuromuscular junction (Enoka and Stuart, 1992). The peripheral modifications have been mainly evaluated by artificial stimulation (i.e., mechanical and EMG evoked responses) of the skeletal muscle and/or motor nerve structures at rest (Martin et al., 2010; Garcia-Vicencio et al., 2016; Maffiuletti et al., 2017).

Aside from contractile mechanisms, the decrease in voluntary and/or electrically induced force with fatigue also depends on the elongation capacity of elastic components, both in series and in parallel, in transmitting force (Ce et al., 2013). Fatigue can increase muscle-tendon complex compliance due to repetitive cycles of long-lasting contractions (Kubo et al., 2001a,b; Zhang and Rymer, 2001) that would lengthen the time for force transmission to the bone due to the inability to store and release elastic energy (Kubo et al., 2001b, 2005). The use of real time ultrasonography has made it possible to assess the displacement of human tendons and single muscle aponeurotic structures during voluntary contractions to determine a muscular stiffness index *in vivo* (Fukashiro et al., 1995; Kubo et al., 2000; Bojsen-Moller et al., 2003). However, these techniques reflect modifications in the stiffness of several structures (muscles, tendons, nerves, and skin) acting around a given joint and not solely stiffness of the muscular tissue. Moreover, conventional ultrasonography techniques often require maximal voluntary contractions (MVC), which may limit its use in clinical cases or acute fatigue states. Shear-wave elastography (SWE) is a very promising alternative which provides reliable and quantitative real-time assessment of specific muscular tissue stiffness at rest, during isometric contractions or passive stretching (Brandenburg et al., 2014; Lacourpaille et al., 2015; Andonian et al., 2016). This technique provides a tissue stiffness index based on tissue shear-wave propagation velocity measurements induced by an acoustic radiation force. The shear-wave propagation velocity directly correlates with the muscle shear elastic modulus (μ) in a homogeneously elastic medium, which is assumed to be the case for muscle tissue (Bercoff et al., 2004; Catheline et al., 2004). Although changes in μ have been well studied after damaging exercise (Lacourpaille et al., 2014, 2017; Guilhem et al., 2016), there is no consensus concerning its modification with fatigue. While some results are in favor of variations of resting μ during fatiguing exercise (Bouillard et al., 2012, 2014; Andonian et al., 2016), some that it is influenced by metabolic changes within the muscle or by peripheral fatigue (Lacourpaille et al., 2017). Thus, the time course of the resting μ with fatigue and its relationship with modifications of neuromuscular indicators of peripheral fatigue is still unknown.

The aim of this study was to investigate the time course of the *vastus lateralis* (VL) muscle resting μ evaluated by SWE and to compare it to the evolution of neuromuscular indicators of peripheral fatigue, (i.e., changes in both voluntary and evoked mechanical and EMG responses), during the repetition of 60 isometric MVCs of KE muscles in healthy humans. We hypothesized that resting VLμ will decrease during this exercise reflecting greater muscle compliance and altered muscle function (greater extent of peripheral fatigue).

## Materials and Methods

### Participants

Fifteen French Army young male soldiers volunteered to participate in the present study. A sample size of 11 (15 subjects included) was deemed sufficient to determine statistical power. This was calculated using the sample size calculator G^∗^Power 3.1.9.2. A large effect size of 0.40 was determined from previous data on the peak knee extensor (KE) torque, alongside a power of 95 % and a significance level of 0.05. Their anthropometrical and physiological characteristics were as follows: age: 29 ± 2.5 years, weight: 79.6 ± 9.1 kg, height: 174 ± 10 cm, BMI: 26.1 ± 1.9 kg^∗^m^−2^, and body fat percentage: 12.5 ± 3.3%. They performed regular physical activities, such as strength training, running, and/or cross training (between 6 and 15 h.w^−1^), with no recent history of muscular, joint, or bone disorders or receiving any medication that could interfere with neuromuscular responses. This study was part of a medico-physiological follow-up performed at the request of the French Army. All the volunteers were fully informed of the experimental procedures, aims, and risks and gave their written assent before any testing was conducted. Each participant participated in an inclusion session consisting of a complete medical examination with anthropometric data collection and complete familiarization with the experimental procedures. This study was approved by the scientific leadership of the French Armed Forces Biomedical Research Institute. All experiments were conducted in accordance with the Helsinki Declaration (Schuklenk, 2001). A version of this manuscript was released as a preprint on bioRxiv at https://doi.org/10.1101/402644 (Siracusa et al., 2018).

### Fatigue Protocol

**FIGURE 1:**
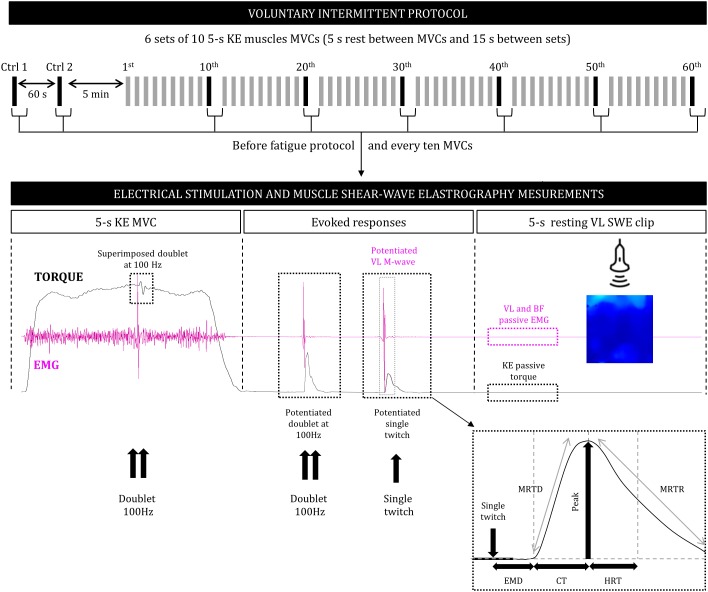
Design of the voluntary intermittent fatigue protocol (top), consisting of a series of voluntary force and electrical stimulation and muscle shear-wave elastography measurements performed before the series and during and after every 10 MVC (bottom). KE, knee extensors; MVC, maximal voluntary contraction; SWE, shear-wave elastography; EMG, surface electromyography; VL, *vastus lateralis*; BF, *biceps femoris*; P, peak torque; EMD, electromechanical delay; CT, contraction time; HRT, half relaxation time; MRTD, maximal rate of torque development; MRTR, maximal rate of torque relaxation.

After a 5-min warm-up at submaximal intensity, participants performed an intermittent voluntary fatigue protocol consisting of 6 × 10 repetitions of alternating isometric 5-s MVCs of the KE muscles and 5-s passive recovery periods between repetitions and 15-s between series (Figure 1). The number of contractions was chosen to generate a high level of voluntary strength loss and peripheral fatigue, as demonstrated previously in adults (Ratel et al., 2015). Double and single electrical stimulations were delivered to the femoral nerve before and every ten MVCs, during the contractions and at rest, to determine the time course of central and peripheral neuromuscular fatigue (see below). Moreover, a linear transducer was fixed to the skin, using a dynamic probe fixation system (USONO, Eindhoven, Netherlands), over the VL muscle and used in SWE mode (musculoskeletal preset) to capture the shear-wave propagation velocity and to determine *in vivo* an index of muscular stiffness. After each ten MVCs and electrical-stimulation series, the resting VL shear elastic modulus (VLμ) was measured during a 5-s period. Participants were not informed of the criterion of task failure (60-MVCs) but had visual feedback of the torque output during the exercise. They were also strongly encouraged by the experimenters during the entire fatiguing task.

### Measurements

#### Anthropometrical Measurements

Body mass was measured to the nearest 0.1 kg using a calibrated scale and height was determined to the nearest 0.01 m using a standing stadiometer. Height and body mass were measured without shoes and while wearing underwear. The percentage of body fat was estimated using skinfold thickness values and Durnin and Womersley (1974) standard equations. Skinfold thickness values were measured to the nearest millimeter, in triplicate, at the biceps, triceps, and subscapular, and suprailiac points on the right side of the body using a Harpenden skinfold caliper (British Indicators, West Sussex, United Kingdom). The same investigator performed all measurements.

#### Femoral Nerve Electrical Stimulation

Evoked contractions of the KE muscles were triggered with a constant-current stimulator (Digitimer DS7A, Hertfordshire, United Kingdom). Single and double square-wave pulses of 1,000 μs, at maximal voltage (400 V), were delivered percutaneously to the femoral nerve using a self-adhesive electrode (10-mm diameter, Ag-AgCl, Type 0601000402, Controle Graphique Medical, Brie-Comte-Robert, France). The cathode was placed in the femoral triangle, 3–5 cm below the inguinal ligament. The anode, a 5 × 10 cm self-adhesive stimulation electrode (Medicompex SA, Ecublens, Switzerland) was placed at the gluteal fold. Small spatial adjustments were initially performed using a ball probe cathode pressed into the femoral triangle to determine the optimal stimulation site. This site corresponded to the position in which the greatest un-potentiated KE single twitch amplitude and concomitant VL compound muscle action potential amplitude were induced. The optimal stimulation intensity, i.e., the intensity at which maximal un-potentiated single twitch and concomitant VL M-wave amplitudes started to plateau, was determined from a progressive recruitment curve. Briefly, simple pulses were induced every 15 s from 40 to 99 mA in 5-mA increments. The supramaximal stimulation intensity ranged from 52 to 91 mA and corresponded to 130% of the optimal intensity.

#### Isometric Maximal Voluntary Contraction

Maximal voluntary and electrically stimulated contractions were assessed under isometric conditions with an isokinetic dynamometer (Cybex Norm, Lumex, Ronkonkoma, NY, United States). Participants were comfortably positioned on an adjustable chair with the hip joint flexed at 30° (0°= neutral position). The dynamometer lever arm was attached 1–2 cm above the lateral malleolus with a Velcro strap. The lever arm was home-built and included a high-density foam pad, placed against the posterior aspect of the leg, and a Velcro strap positioned over the anterior aspect of the leg. This configuration was chosen to reduce cushioning and improve torque transmission and resolution, which is critical when evaluating twitch contractile properties and the voluntary activation level (Gandevia, 2001). The axis of rotation of the dynamometer was aligned with the lateral femoral condyle of the femur. The participants were secured firmly with Velcro straps across the hips and chest to minimize upper body movement. During each MVC, participants were instructed to grip the seat to stabilize the pelvis. Before the voluntary fatigue protocol, two 5-s MVCs of the KE were performed, with 60-s passive recovery periods. The absolute MVC peak torque was determined as the peak force reached during maximal efforts and was defined as the control “non-fatigued” values. All measurements were taken from the participant’s dominant leg (right leg for all participants), which was fixed at 90° (0°= knee fully extended). This muscle length was selected to ensure a good reliability of the neuromuscular assessments. Indeed, it is a length close to the optimal angle of force generating. This allowed us to induce a greater extent of peripheral fatigue and to detect easily muscle stiffness modifications compared to short muscle lengths. Torque data was corrected for gravity using Cybex software at an angle of 45° and was acquired and digitized on-line at a rate of 2 kHz by an A/D converter (Powerlab 8/35, ADInstruments, NSW, Australia) driven by Labchart 8.0 Pro software (ADInstruments).

#### Electrically Evoked Torque and Maximal Voluntary Activation Level

The double pulse (at 100 Hz) superimposition technique, based on the interpolated-twitch method (Merton, 1954), enabled us to estimate the maximal KE voluntary activation level (VAL). Briefly, superimposed (Db_s100Hz_) and potentiated (Db_pot100Hz_) double stimulations were delivered during MVC after the torque had reached a plateau and 3-s after cessation of the contraction, respectively. This allowed us to obtain a potentiated mechanical response and hence reduce the variability of the VAL values (Kufel et al., 2002). The superimposed doublet was preferred to a superimposed single twitch (T_w_) because it results in a greater signal-to-noise ratio and thus allows the detection of small changes in VAL (Gandevia and McKenzie, 1988). The ratio of the amplitude of the Db_s100Hz_ over that of the Db_pot100Hz_ for the relaxed muscle (control doublet) was then calculated to obtain the VAL, (i.e., indicator of central fatigue), as follows:

$$\text{VAL }(\%)=[1-\left(\frac{\text{Dbs}100\text{Hz}}{\text{Dbpot}100\text{Hz}}\right)\text{ x }100]$$

After cessation of the contraction and 3-s after the Db_pot100Hz_ a single T_w_ were delivered to the relaxed muscle in a potentiated state (T_wpot_; 3-s between; Figure 1; Millet et al., 2002). This set of measurements (MVC with superimposed doublet + evoked stimuli to the relaxed muscle) was repeated before and after the fatigue protocol and every ten MVCs. Then, the following parameters were obtained from the T_wpot_ response: peak torque (P), electromechanical delay (EMD), contraction time (CT), half relaxation time (HRT), maximal rate of torque development (MRTD), i.e., the maximal value of the first derivative of the mechanical signal divided by the peak torque, maximal rate of torque relaxation (MRTR), i.e., the maximal value of the first derivative of the mechanical signal divided by the peak torque.

#### EMG Activity

The EMG signals of the VL and *biceps femoris* (BF) muscles were recorded, during voluntary and evoked contractions, using bipolar silver chloride surface electrodes (Blue Sensor N-00-S, Ambu, Denmark). The recording electrodes were taped lengthwise to the skin over the muscle belly, as recommended by SENIAM (Hermens et al., 2000), with an inter-electrode distance of 20 mm. The reference electrode was attached to the patella. Low impedance (*Z* < 5 kΩ) at the skin-electrode surface was obtained by shaving, gently abrading the skin with thin sand paper, and cleaning with alcohol. EMG signals were amplified (Dual Bio Amp ML 135, ADInstruments, Australia) with a bandwidth frequency ranging from 10 to 500 Hz (common mode rejection ratio > 85 dB, gain = 1,000) and simultaneously digitized together with the torque signals. The sampling frequency was 2 kHz. During the fatigue protocol, the root mean square (RMS) values of the VL EMG activity were calculated during the MVC trials over a 0.5-s period after the torque had reached a plateau and before the superimposed stimulation was evoked. This RMS value was then normalized to the maximal peak-to-peak amplitude of the potentiated VL M-wave (RMS × M_max_^−1^).

#### Shear-Wave Elastography

An ultrasound scanner (Aixplorer version 12.2; Supersonic Imagine, Aix-en-Provence, France) coupled with a linear transducer array (4–15 MHz, SuperLinear 15-4; Vermon, Tours, France) was used in SWE mode (musculoskeletal preset), as previously described (Bercoff et al., 2004). Briefly, The SWE technique is based on ultrafast ultrasound sequences that are performed to capture shear-wave propagation. The SWE technique relies on the acoustic radiation force to remotely generate low-frequency shear-waves in tissues, (e.g., muscle, breast, liver), and can be achieved using the same piezoelectric arrays as those used in conventional ultrasonic scanners (Bercoff et al., 2004). The shear-wave displacement field is saved via one-dimensional cross-correlation of consecutive radio frequency signals along the ultrasound beam axis as a function of time. The shear-wave speed is then calculated in each pixel of the resulting image using a time-of-flight algorithm on the displacement movies. The shear-wave propagation velocity, typically a few meters per second in soft tissues, correlates directly with the muscle shear elastic modulus (μ) if the medium is assumed to be purely elastic, which is well accepted in muscle elastography studies (Bercoff et al., 2004; Catheline et al., 2004). The μ was obtained as follows:

$$\mu =p\;x\;{\mathrm{Vs}}^{2}$$

where *p* is the muscle density (1,000 kg^∗^m^−3^) and V_s_ is the shear wave speed (in m^∗^s^−1^). This equation implicitly neglects viscous effects. Studies revealed that the shear-wave velocity is almost independent of the frequency of the mechanical shock when measured longitudinally by SWE, demonstrating no significant viscous effects (Catheline et al., 2004).

**FIGURE 2:**
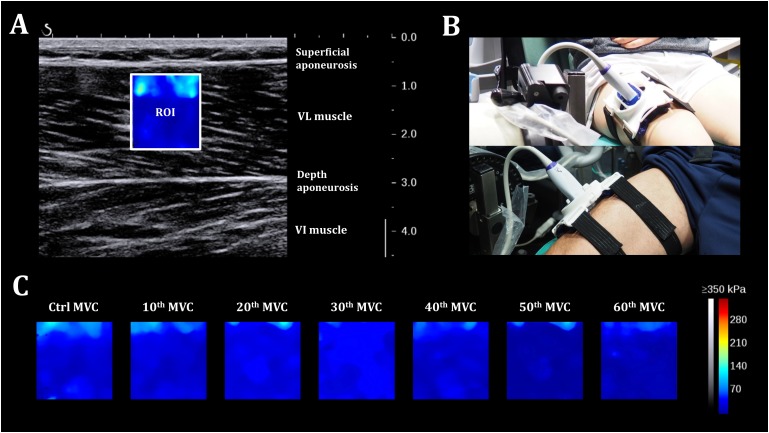
Shear-wave elastography. **(A)** 2D real-time color map of the shear elastic modulus in the *vastus lateralis* muscle obtained at 1 Hz with a spatial resolution of 1 × 1 mm. ROI: region of interest. **(B)** Participant positioning on the isokinetic ergometer with the probe fixation system placed over the right thigh. **(C)** A representative scheme of evolution of the 2D color map muscle shear elastic modulus for one individual during the fatigue test.

Shear-wave elastography measurements were carefully standardized by fixing the ultrasound probe using a dynamic probe fixation system (360° adjustment) placed over the skin and coating it with a water-soluble transmission gel (Aquasonic, Parker laboratory, Fairfield, NJ, United States) to improve acoustic coupling. The B-mode ultrasound was first set to determine the optimal probe location and maximize the alignment between the transducer and the direction of the muscle fascicles. Probe alignment was considered to be correct when VL muscle fascicles and aponeurosis could be delineated without interruption across the image. A probe orientated in parallel to the muscle fascicles provided the most reliable muscle elasticity measurements (Gennisson et al., 2010). After probe positioning, a fixed-size square region of interest (ROI; ∼1.5 cm^2^), i.e., a region in which shear-wave propagation was analyzed within the muscle, was placed in the middle of the B-mode image below the superficial aponeurosis within the VL muscle. A 2D real-time color map of the shear elastic modulus was then obtained at 1 Hz with a spatial resolution of 1 × 1 mm (Figure 2A). Finally, a 5-s clip was performed when the 2D real-time color map was maximally homogeneous to avoid any influence of the previous muscle contraction. During scans, the knee joint was positioned at 90° (0°= knee fully extended) and participants were asked to stay as relaxed as possible. Passive KE torque and VL and BF EMG activity were continuously and carefully monitored to verify this relaxed position.

The resting μ values were averaged over the largest ROI and the average of the five consecutive images (five values were recorded at one sample per second), obtained during the clip, was used for subsequent analyses. Reproducibility of the scans was also determined for each measurement. DICOM images were then transferred to a workstation and analyzed using a MATLAB script developed in our laboratory (MathWorks, Natick, MA, United States).

The VL muscle was chosen because studies showing decreases in muscle stiffness by assessing aponeurosis or muscle tendon junction displacements after repeated isometric contractions were made exclusively in the VL muscle (Kubo et al., 2001a,b). It will allow future comparisons with the present study regarding single muscle stiffness. The other quadriceps heads were not assessed in this study by methodological/schedule reasons.

### Statistical Analysis

The data were screened for normality of the distribution and homogeneity of variances using the Shapiro–Wilk normality and Levene tests, respectively. Differences in absolute values and relative changes were analyzed by one-way ANOVA (effect: number of repetitions) with repeated measures. If the ANOVA revealed significant effects or interactions between factors, Fisher’s LSD *post hoc* test was applied to test the discrimination between means. The intrasession repeatability of the resting shear elastic modulus was evaluated for the VL muscle between each five measurements, obtained during the 5-s movie, by calculating the intraclass correlation coefficient (ICC) and standard error of measurement (SEM) (Hopkins, 2000). Results with a *P*-value < 0.05 were considered to be significant. Statistical procedures were performed using Statistica 8.0 software (Statsoft Inc, United States). The results are presented in means ± SD for table and text and means ± SEM for figures.

## Results

### Maximal Voluntary Torque

**FIGURE 3:**
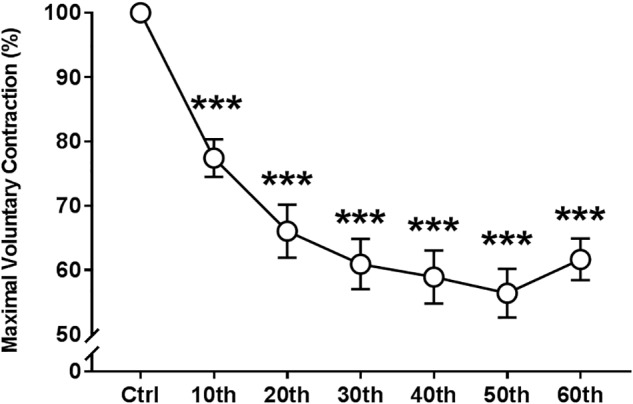
Time course of the maximal voluntary contraction of the knee extensor muscles during the fatigue protocol. Mean ± SEM. ^∗∗∗^Significant differences from control (Ctrl) values (*P* < 0.001).

ANOVA revealed significant absolute and relative (to the control “non-fatigued” value) repetition-dependent effects on MVC torque (*P* < 0.001, Figure 3). MVC decreased by 38.4 ± 12.6% by the end of the exercise (*P* < 0.001), with a significant reduction starting from the 10th MVC (*P* < 0.001).

### VL Resting Shear Elastic Modulus

The measurements were reproducible (five measurements obtained during a 5-s clip) throughout the fatigue protocol. The ICC (95% CI) and SEM (kPa) values were as follows for each measurement: Control (99.5%, 0.2 kPa), MVC10 (99.5%, 0.24 kPa), MVC20 (98.9, 0.27 kPa), MVC30 (99.0%, 0.26 kPa), MVC40 (96.7%, 0.27 kPa), MVC50 (99.0%, 0.26 kPa), and MVC60 (97.5%, 0.26 kPa).

**FIGURE 4:**
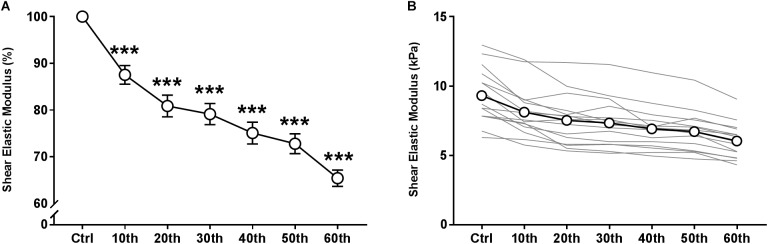
Time course of resting shear elastic modulus of the *vastus lateralis* muscle over the fatigue protocol. **(A)** relative means values and **(B)** individual (thin gray lines) and mean (white dots and bold black line) absolute values. Mean ± SEM. ^∗∗∗^Significant differences from control (Ctrl) values (*P* < 0.001).

The time course of the relative and absolute resting VLμ (in % and kPa, respectively) is presented in Figure 4A,B. A representative scheme of the kinetics of the 2D color map of the muscle μ is presented in Figure 2C. ANOVA revealed a significant repetition-dependent effect for both absolute and relative resting VLμ (*P* < 0.001). Relative resting VLμ values significantly decreased progressively from the 10th MVC (−12.4 ± 7.7%, *P* < 0.001) to the end of the exercise (−34.7 ± 6.7%, *P* < 0.001).

### Peripheral Mechanisms of Fatigue

#### Electrically Stimulated Potentiated Torque

**FIGURE 5:**
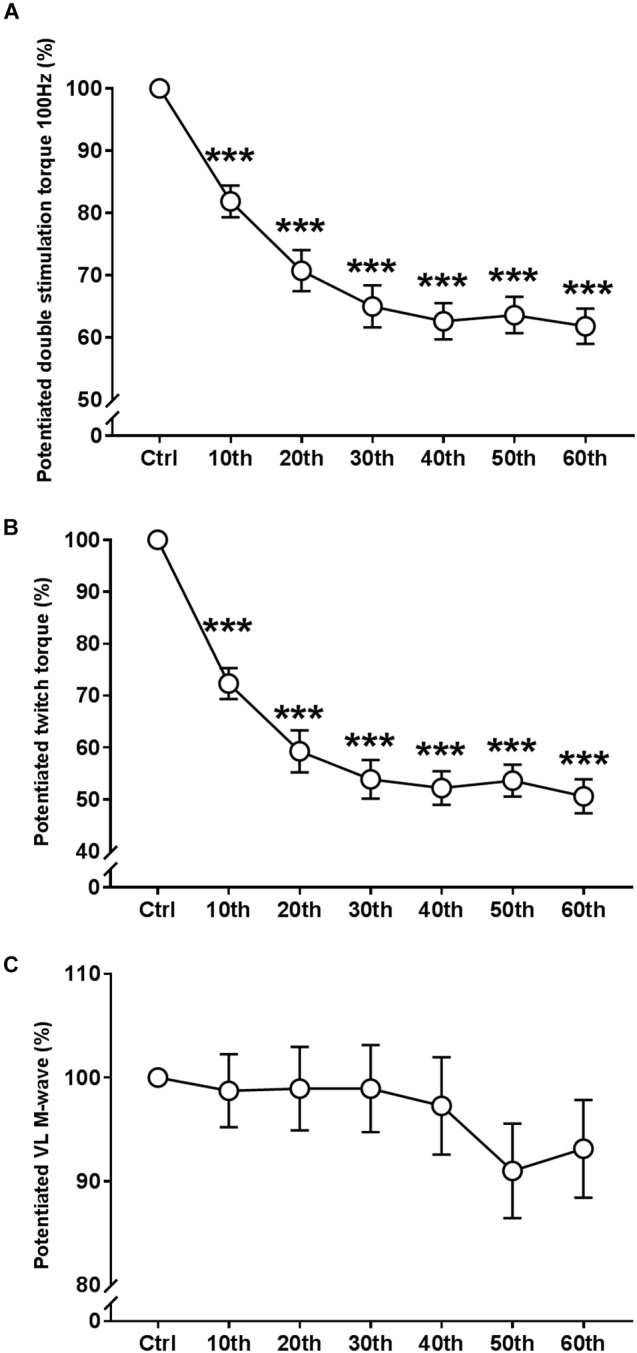
Time course of the evoked mechanical and EMG responses during the fatigue protocol. **(A)** Evolution (%) of the potentiated double stimulation torque of the knee extensor muscles (100 Hz). **(B)** Evolution (%) of the potentiated twitch torque of the knee extensor muscles. **(C)** Evolution (%) of the potentiated M-Wave of the *vastus lateralis* (VL) muscle. Mean ± SEM. ^∗∗∗^Significant differences from control (Ctrl) values (*P* < 0.001).

ANOVA revealed a significant repetition-dependent effect for both absolute and relative Db_pot100Hz_ and T_wpot_ torque similar to that of MVC (*P* < 0.001, Figure 5A,B). Db_pot100Hz_ and Tw_pot_ torque decreased by 38.2 ± 10.8% and 49.4 ± 12.6% by the end of the exercise, respectively (*P* < 0.001). Reductions in both relative Db_pot100Hz_ and T_wpot_ torque values started from the 10th MVC (*P* < 0.001 for both), similarly to MVC.

#### Potentiated *vastus lateralis* M-Wave Amplitude

There were no significant changes in the potentiated VL M_max_ amplitude during the entire fatigue protocol (Figure 5C and Table 1).

**Table 1:** Time course of absolute neuromuscular and elastographic values.

	Ctrl	MVC10	MVC20	MVC30	MVC40	MVC50	MVC60
KE MVC (Nm)	352 ± 68	270 ± 53^∗∗∗^	226 ± 49^∗∗∗^	208 ± 46^∗∗∗^	202 ± 48^∗∗∗^	192 ± 38^∗∗∗^	211 ± 39^∗∗∗^
**Nervous factors**							
KE VAL (%)	89.0 ± 3.9	87.7 ± 8.3	82.8 ± 12.7	80.9 ± 12.5	75.9 ± 21.9	79.1 ± 13.9	81.2 ± 12.3
VL RMS/M_pot (au)	0.06 ± 0.02	0.06 ± 0.02	0.06 ± 0.03	0.05 ± 0.02	0.05 ± 0.02	0.06 ± 0.02	0.07 ± 0.02
**Muscular factors**							
*Evoked response*							
KE Db100Hz_pot (Nm)	114.3 ± 14.3	94.0 ± 18.9^∗∗^	81.6 ± 22.3^∗∗∗^	75.0 ± 21.1^∗∗∗^	71.9 ± 17.6^∗∗∗^	73.2 ± 17.9^∗∗∗^	70.8 ± 16.0^∗∗∗^
KE Tw_pot (Nm)	77.9 ± 10.2^∗∗∗^	56.5 ± 13.1^∗∗∗^	46.7 ± 15.6^∗∗∗^	42.4 ± 14.3^∗∗∗^	41.1 ± 12.7^∗∗∗^	42.0 ± 11.8^∗∗∗^	39.6 ± 11.7^∗∗∗^
Peak-to-peak amplitude	5.2 ± 2.8	5.2 ± 2.7	5.2 ± 2.7	5.1 ± 2.7	5.1 ± 2.7	4.8 ± 2.6	4.8 ± 2.5
*Mechanical properties of twitch*							
EMD (ms)	28.8 ± 10.8	32.0 ± 12.3	32.1 ± 12.7	33.2 ± 12.7	33.1 ± 13.1	33.9 ± 12.8	35.0 ± 12.8
CT (ms)	47.7 ± 9.5	50.6 ± 9.7	52.2 ± 10.8	52.8 ± 11.2^∗^	54.0 ± 11.6^∗^	56.0 ± 12.8^∗^	57.5 ± 12.9^∗^
MRTD (Nm.ms^−1^)	4.8 ± 3.3	3.8 ± 3.1	2.7 ± 1.9^∗^	2.4 ± 1.6^∗∗^	2.3 ± 1.7^∗∗^	2.3 ± 1.5^∗∗^	2.2 ± 1.4^∗∗^
HRT (ms)	128.2 ± 39.7	121.3 ± 42.9	112.6 ± 34.5	110.8 ± 33.7	110.8 ± 38.8	107.6 ± 38.9	104.8 ± 34.0
MRTR (Nm.ms^−1^)	4.4 ± 2.9	3.5 ± 2.4	3.0 ± 2.4	2.7 ± 2.2	2.4 ± 1.9	2.5 ± 2.0	2.2 ± 1.8
*Shear-wave elastography*							
Shear elastic modulus (kPa)	9.3 ± 2.0	8.1 ± 1.8	7.5 ± 1.8^∗∗∗^	7.4 ± 1.7^∗∗∗^	6.9 ± 1.5^∗∗∗^	6.7 ± 1.4^∗∗∗^	6.1 ± 1.3^∗∗∗^
**Rest neuromuscular outcomes**							
KE passive torque (Nm)	0.4 ± 0.1	0.6 ± 0.9	0.8 ± 0.9	0.6 ± 0.5	0.7 ± 0.5	0.8 ± 1.2	0.6 ± 0.7
VL passive RMS (mV)	0.004 ± 0.005	0.004 ± 0.005	0.004 ± 0.004	0.004 ± 0.003	0.003 ± 0.003	0.003 ± 0.003	0.003 ± 0.003
BF passive RMS (mV)	0.002 ± 0.001	0.002 ± 0.001	0.002 ± 0.001	0.002 ± 0.000	0.002 ± 0.001	0.002 ± 0.000	0.002 ± 0.000

*Mean ± SD. KE, knee extensors; MVC, maximal voluntary contraction; VL, vastus lateralis; BF, biceps femoris; VAL, voluntary activation level; RMS, root mean square; M_pot, potentiated M-wave; Db100Hz_pot, double pulses induced by electrical stimulation; Tw_pot, single pulse induced by electrical stimulation; EMD, electromechanical delay; CT, contraction time; MRTD, maximal rate of torque development; HRT, half relaxation time; MRTR, maximal rate of torque relaxation. ^∗^P < 0.05, ^∗∗^P < 0.01, and ^∗∗∗^P < 0.001. Significant differences from control (Ctrl) values.*

#### Electro-Mechanical Properties of Single Twitch Torque

**FIGURE 6:**
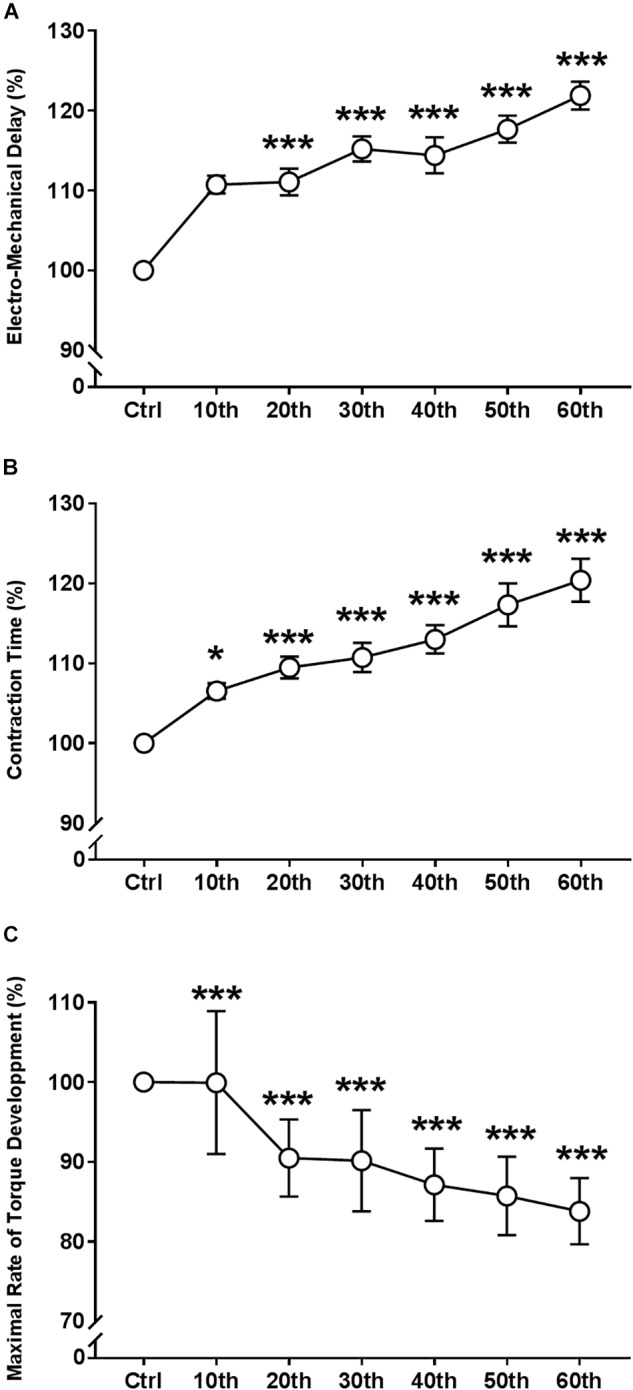
Time course of the electro-mechanical properties of single twitch torque during the fatigue protocol. **(A)** Evolution (%) of the electro-mechanical delay. **(B)** Evolution (%) of the contraction time. **(C)** Evolution (%) of maximal rate of torque development. Mean ± SEM. Significant differences from control (Ctrl) values: ^∗^*P* < 0.05 and ^∗∗∗^*P* < 0.001.

ANOVA revealed a significant repetition-dependent effect for the absolute CT (*P* < 0.001), but not for EMD (Table 1). However, relative EMD and CT significantly prolonged during the fatigue protocol, reaching 21.9 ± 6.8% and 20.4 ± 10.4% of their initial values, respectively, (*P* < 0.001 for both; Figure 6A,B) by the end of the exercise. Moreover, the absolute and relative MRTD significantly decreased by the end of the exercise: 51.0 ± 12.7% (*P* < 0.001; Figure 6C). Both absolute and relatives MRTR and HRT values were not modified with fatigue.

### Central Mechanisms of Fatigue

#### Voluntary Activation Level and EMG Activity

Absolute VAL and RMS⋅M_max_^−1^ ratio of the VL muscle values are shown in Table 1. There were no significant changes during the entire fatigue protocol.

#### Passive EMG Activity and Torque

Passive KE torque and VL and BF EMG activity assessed during the SWE measurements remained unchanged during the entire fatigue protocol (Table 1).

## Discussion

In agreement with our hypothesis, absolute resting VLμ significantly decreased during the fatigue protocol from the 20th MVC to the end of the exercise (60th MVC) for all participants, with the loss ranging from −23 to −45%. The neuromuscular indicators of peripheral fatigue were also altered with fatiguing exercise (both voluntary and evoked torque). Changes in the single twitch electromechanical properties (EMD, CT, and MRTD) were also observed at the end of test.

### Reproducibility of Measurements

Shear-wave elastography has been demonstrated to be a highly promising alternative to conventional elastography techniques, and provides a reliable and quantitative real-time assessment of muscular tissue stiffness (Brandenburg et al., 2014; Lacourpaille et al., 2015; Andonian et al., 2016). This study is the first to address the interest of μ on a non-contracted muscle (VL) at various stages of the fatigue during a series of isometric MVC of the KE muscles. Methodological factors such as slight probe motion, compression, variability of the measurement, and the ability of subjects to achieve a fully relaxed state were meticulously controlled to ensure good reliability of the measurements (Lacourpaille et al., 2012; Eby et al., 2013). There was little variability of the measurements (five images obtained during the 5-s clip) during the entire fatigue protocol. ICC and SEM values varied from 96.7% to 99.5% and from 0.20 to 0.26 kPa for the VL muscle, despite the development of fatigue. We achieved this level of reproducibility by positioning a dynamic probe fixation system over the skin onto the VL muscle (Figure 2B) avoiding any muscle deformation. In addition, the EMG VL and BF EMG activity and passive torque did not change during the SWE recordings (Table 1), verifying that the participants were in a relaxed state.

Our resting VLμ baseline values are concordant with existing published SWE data (Lacourpaille et al., 2017). The mean and standard deviation of 9.3 ± 2.0 kPa (ranging from 6.3 to 12.9 kPa) for a knee flexion of 90° (0°= knee fully extended) are in accordance with the values of <10 kPa for the quadriceps muscles for the same knee flexion (90°) in active individuals in a non-fatigued state (Lacourpaille et al., 2017). However, our control values were higher than those of other studies (∼3–5 kPa) for the VL muscle (Lacourpaille et al., 2012; Botanlioglu et al., 2013; Andonian et al., 2016). These differences may be explained by the short muscle length used in these studies (knee fully extended), as it is known that resting μ and muscle stiffness increase with increasing muscle length (Lacourpaille et al., 2014, 2017).

### Muscle Stiffness During and Immediately After Fatiguing Exercise

Most previous studies have characterized fatigue or training-induced changes in muscle-tendon stiffness by exploring the movement of human tendons and/or aponeurosis structures *in vivo* (Fukashiro et al., 1995; Kubo et al., 2000; Bojsen-Moller et al., 2003). Several studies have shown greater muscle-tendon compliance (increases in the elongation of connective structures for the same level of the produced force) after repeated contractions of the KE, (e.g., VL muscle), and arm muscles (Vigreux et al., 1980; Kubo et al., 2001a,b). However, one of the main limitations of these studies is that ultrasound-based techniques reflect modifications in the stiffness of several structures (muscles, tendons, nerves and skin) around a given joint and are not specific to skeletal muscle stiffness.

As mentioned above, SWE provides a quantitative and reliable measurement of individual muscular tissue stiffness (Brandenburg et al., 2014; Lacourpaille et al., 2015; Andonian et al., 2016). A strong linear relationship between individual muscle force and dynamic muscle μ, evaluated during contractions, has been demonstrated, suggesting that it may be a good index of individual force (Bouillard et al., 2011). Furthermore, Bouillard et al. (2012) confirmed this relationship during submaximal isometric contractions, even when muscle fatigue occurs. These results suggest that SWE can be used to quantify relative modifications in voluntary force, even during fatiguing conditions. In another study (Bouillard et al., 2014), the same authors showed that when fatigue was previously induced in one quadriceps muscle (VL), lower dynamic μ values were observed, both initially and during a subsequent submaximal isometric task, relative to the control non-fatigued muscle. Although the amplitude of the muscle μ appears to be affected by fatigue, the underlying mechanisms for its decline are still unknown.

In our study, we observed a progressive decrease in the absolute resting μ of the VL muscle from the 20th MVC (−12.4 ± 7.7%) to the end of the fatiguing exercise (−34.7 ± 6.7%), suggesting a progressive rise in muscle compliance (at 50% of the VL muscle length). Some studies have suggested that increases in muscle-tendon compliance can be explained, in part, by alterations of the viscoelastic properties of the intramuscular connective tissue due to repeated contractions (Warren et al., 1971; Vigreux et al., 1980). Currently, specific viscoelastic properties of the soft tissues may be quantified by real-time supersonic shear imagining, as described in the literature (Bercoff et al., 2004; Gennisson et al., 2010), but no study has investigated the effects of peripheral fatigue on the viscoelastic properties of skeletal muscle and its role in force transmission.

The decline of the resting μ of the VL muscle found in our study is consistent with those observed for locomotor muscles after strenuous long-distance running, evaluated by invasive techniques, such as tension-myography and muscle belly deformation (Garcia-Manso et al., 2011; Giovanelli et al., 2016), as well as SWE (Andonian et al., 2016). However, these exercise-models are clearly very different from that used in our study and the extent of induced fatigue may be different than that in our model. For example, Andonian et al. (2016) observed significant decreases in the resting μ of the quadriceps muscles (without distinguishing between the heads, but mainly in the VL muscle) after an extreme mountain ultra-marathon, which were still reduced after more than 45 h. However, the relationship between changes in muscle μ and neuromuscular parameters of fatigue was not explored. In contrast to these results, Akagi et al. (2017) observed an increase in resting μ (∼+7%) of the *medial gastrocnemius*, but not *soleus* or *lateral gastrocnemius,* muscle using another model of fatiguing exercise (sustained 10% MVC during 1 h). Moreover, Lacourpaille et al. (2017) showed that intense, non-damaging exercise (3 × 10 concentric MVCs at 120^∘∗^s^−1^) did not modify the resting μ of the elbow flexor muscles. They suggested that the resting μ would not be influenced by peripheral factors originating from fatiguing contractions, contrary to those observed in our study. However, in their study 30 concentric MVCs did not induce a significant decrease in voluntary torque, limiting the conclusions concerning the relationship between fatigue and changes in resting μ. It is possible that in our study the significant decline in resting VLμ during the fatigue protocol may be closely related to the greater observed extent of exercise-induced peripheral fatigue.

### Shear Elastic Modulus, Peripheral Fatigue Outcomes and Force Transmission Properties

The mechanisms underlying changes in muscle μ by SWE after fatiguing exercise are not well described. As expected, in our study the significant reductions in the KE MVC were mostly explained by alterations of peripheral factors. The large extent of peripheral fatigue observed was mostly reflected by a significant decrease in the amplitude of the single- and double-stimulated responses at end of the exercise. These results may suggest modification of the excitation-contraction coupling (Bigland-Ritchie, 1981; Edwards, 1981; Enoka and Stuart, 1992; Gandevia, 2001; Debold, 2012; Millet et al., 2012), more specifically, impairments in calcium homeostasis. Moreover, the EMD (+21.9 ± 6.8%) and CT (+20.4 ± 10.4%) increased significantly until the end of the exercise and the MRTD (−51.0 ± 12.7%) was lower than control values. These results are suggesting that any alteration on the electromechanical properties of the skeletal muscle are mainly associated with elastic rather than electrochemical processes, [e.g., alteration in the propagation of action potentials along the muscle membrane (Ce et al., 2013, 2015)], due to the lack of modification in the potentiated VL M-wave amplitude during the fatigue protocol. Under fatiguing conditions, changes in electromechanical properties of the skeletal muscle would result in a longer time to stretch a more elastic muscle and to transfer tension to the tendon insertion point (Zhou et al., 1995, 1998). These findings could suggest a relationship between the decline in resting VLμ and alteration of quadriceps muscle elastic properties (higher muscle compliance) with fatigue. For instance, studies have suggested that fatigue-induced modification of the viscoelastic properties in the muscle-tendon complex could be strongly associated with a longer EMD and higher CT (mechanical component) (Taylor et al., 1997; Zhang and Rymer, 2001). However, no findings allow us to determine this association in this study limiting conclusions. Thus, in the present study one head of the quadriceps muscle complex was evaluated limiting therefore interpretations about changes of the whole quadriceps neuromuscular properties with fatigue. The evaluation of the viscoelastic properties of muscles could help us to better understand the higher muscle compliance experienced after fatiguing isometric contractions. However, modification of the viscoelastic properties of skeletal muscle due to fatigue is yet to be investigated by supersonic imaging approaches.

### Main Limitations of This Study

This study provides scientific evidence related to changes of VL muscle μ and peripheral fatigue. However, it presents some methodological limitations: (i) in the present study the whole quadriceps complex was not evaluated limiting interpretation of our results concerning others synergist muscles (*rectus femoris* and *vastus medialis*) and their association with the whole quadriceps neuromuscular assessments. A recent study (Heales et al., 2018) indicated that muscle stiffness of RF, VL and VM was not similarly affected after series of eccentric contractions since it only increased in RF. These results may indicate that biarticular muscles such as RF are more affected by a damaging exercise than their monoarticular synergists (VL and VM). Then, (ii) it is possible that the magnitude of changes in muscle stiffness was different at other muscle lengths. Indeed, muscle stiffness was higher at long compared to short muscle lengths after eccentric contractions (Lacourpaille et al., 2014). However, the relationship between muscle fatigue, stiffness and muscle length need therefore to be more investigated.

Finally, given the characteristics of the population (well-trained young male soldiers) and the fatigue protocol (maximal isometric contractions), the results of the present study must be cautiously interpreted. It is possible that our results cannot be transferred to other populations (females, untrained men, older volunteers) or mode of contraction. Muscle fatigue is a complex phenomenon and variables such as sex (Hunter, 2009), age (Ratel et al., 2015), pathology (Garcia-Vicencio et al., 2016), task (Enoka and Stuart, 1992) have been shown to affect the magnitude of strength loss and the time of course of central and peripheral mechanisms. This intrinsic variability may have an important effect on the magnitude of the shear elastic modulus when fatigue is installed.

## Conclusion

This study describes the time course of the resting VLμ in parallel to changes in both voluntary and electrostimulated torque amplitudes and electromechanical properties of the single twitch during the repetition of maximal voluntary fatiguing exercise of the KE muscles in young physically active men. Changes in the resting VLμ could reflect a decline in muscle function explained. In this study, the progressive decrease of the resting VLμ (i.e., higher muscle compliance) experienced during the fatiguing exercise could directly affect the force transmission capabilities accounting in partly for peripheral fatigue. However, the mechanism underlying the decrease in resting μ under fatiguing conditions still needs to be investigated. The study of both the muscle and tendon characteristics during and after fatiguing exercises in the context of sports medicine or the military is essential for the prevention of over-use injuries resulting from repeated exposure to low or high levels of force.

## Data Availability

The datasets generated for this study are available on request to the corresponding author.

## Ethics Statement

This study was part of a medico-physiological follow-up performed at the request of the French Army. All the volunteers were fully informed of the experimental procedures, aims, and risks and gave their written assent before any testing was conducted. This study was approved by the scientific leadership of the French Armed Forces Biomedical Research Institute. All experiments were conducted in accordance with the Helsinki Declaration (Schuklenk, 2001).

## Author Contributions

JS, KC, and SG-V designed the study, performed the analysis and interpretation of the data, and drafted the manuscript. JS, KC, CB, SC, P-ET-D, AM, and SG-V participated in the data collection. P-ET-D, AM, and CB critically revised the manuscript. All authors gave their final approval of this version of the manuscript for publication.

## Conflict of Interest Statement

The authors declare that the research was conducted in the absence of any commercial or financial relationships that could be construed as a potential conflict of interest.
